# Impact of driver licensing renewal policies on older driver crash involvement and injury rates in 13 states, 2000–2019

**DOI:** 10.1186/s40621-024-00555-9

**Published:** 2025-01-14

**Authors:** Cara J. Hamann, Jonathan A. Davis, Gilsu Pae, Motao Zhu, Gregory H. Shill, Brian Tefft, Joseph E. Cavanaugh

**Affiliations:** 1 Injury Prevention Research Center, University of Iowa, 145 N Riverside Dr., Iowa City, IA 52242 USA; 2 Department of Epidemiology, University of Iowa College of Public Health, 145 N Riverside Dr., Iowa City, IA 52242 USA; 3 Department of Occupational and Environmental Health, University of Iowa College of Public Health, 145 N Riverside Dr., Iowa City, IA 52242 USA; 4 Department of Epidemiology, College of Public Health, The Ohio State University, 700 Children’s Drive, Columbus, OH 43205 USA; 5 Center for Injury Research & Policy, Nationwide Children’s Hospital, 700 Children’s Drive, Columbus, OH 43205 USA; 6 College of Law, University of Iowa, 280 Boyd Law Building, Iowa City, IA 52242 USA; 7 AAA Foundation for Traffic Safety, 607 14th Street NW, Washington, DC 20005 USA; 8 Department of Biostatistics, University of Iowa College of Public Health, 145 N Riverside Dr., Iowa City, IA 52242 USA

**Keywords:** Motor vehicle, Collision, Laws, Traffic, Aging

## Abstract

**Background:**

Motor vehicle crashes are the second leading cause of injury death among adults aged 65 and older in the U.S., second only to falls. A common state-level approach to mitigating older adult crash risk is the implementation of driver license renewal policies which vary largely between states and data on their effectiveness in preventing crashes and injuries are limited. To fill this gap, the aim of this study is to examine the association between state driver license renewal policies and older driver crash and injury rates.

**Methods:**

Historical crash data, license renewal policy data, and other relevant policy and demographic data were gathered from 13 U.S. states (CO, IL, IA, KS, MN, MO, NE, ND, OH, SD, UT, WI, WY) for years 2000 through 2019, inclusive. Main exposures included six license renewal policies: renewal period, in-person renewal frequency, vision testing, knowledge testing, on-road drive testing, and mandatory physician reporting. The primary outcomes were crash and injury rates per 100,000 population.

**Results:**

The study population included 19,010,179 crash-involved drivers aged 40 and older. State policies became less restrictive in many states over the study period, even for drivers aged 75 and older, resulting in longer times between renewals and fewer in-person renewal requirements. Loosening of in-person renewal from every time to less than every time was associated with increased crash rates, among drivers aged 65 to 74 (RR_crash_ = 1.08, 95% CI: 1.01–1.16). A longer duration between in-person renewals was associated with increased injury rates among drivers 75 and older (RR_injury_ = 1.18, 95% CI: 1.00–1.39).

**Conclusions:**

Generally, state policies became less restrictive and resulted in longer required intervals between license renewal. Loosening of driver license renewal policies was associated with increased crash and injury rates. However, safety benefits of restrictive older driver licensing policies should be carefully weighed against costs to older adult well-being and quality of life following licensure loss. Additional methods to assess fitness to drive are necessary to identify the mechanisms behind the increased rates.

## Background

Motor vehicle crashes are the second leading cause of injury death among adults aged 65 and older in the U.S., second only to falls [1]. Annually, there are more than 160,000 older adults (aged 65 +) seen in emergency departments due to motor vehicle crash-related injuries [2]. Drivers aged 70 and older have elevated crash and injury rates per mile driven and worse injury recovery compared to middle-aged drivers [3, 4]. The proportion of older drivers has been increasing as the U.S. population continues to lives longer and remain mobile longer [5, 6]. The increased number of older drivers could impact crash and injury outcomes, especially in the absence of effective interventions.

However, given the auto-centricity and car dependence of the U.S., the ability to drive is important and often necessary for sustained mobility among older adults [7, 8]. Transportation mobility is essential for older adults to maintain their independence, social connectedness, access to services (e.g., healthcare), and overall quality of life [9–11].

A common state-level strategy to increase road safety and assess driving fitness is through driver license renewal policies related to vision testing, in-person renewal requirements, license renewal periods, knowledge and driving tests, and mandatory physician reporting [12]. Despite the commonality of these policies there is a considerable variation in the parameters of each policy between states [12]. Additionally, the evidence of the laws’ effectiveness in curbing crashes and injuries is limited and mixed [12].

One study published in 2014 examined driver license renewal policies in relation to fatal crashes and found in-person renewal and vision testing requirements were effective in reducing fatal crash involvement rates among drivers aged 85 and older [13]. The other age groups and licensing policies they examined had little association with fatal crash rates. However, a recent study based in Utah that examined a new law requiring mandatory vision testing at each license renewal for drivers aged 65 and older did not find reductions in injury or fatality rates relative to drivers aged 45 to 64 [14]. There has been little further research related to driver license renewal policy effectiveness in terms of more recent analyses focused and analyses examining impacts on crash and non-fatal injury rates (as opposed to fatal crash rates only). This study aims to fill gaps related to understanding the impact of driver license renewal policies on older driver crash and injury rates by examining 20 years of crash data from 13 states within the north-central U.S..

## Methods

### Data sources and study population

Data for this study included police-reported crash data, state driver license renewal policies, state traffic safety factors, environmental and community factors. Crash data were drawn from 13 states (Colorado, Illinois, Iowa, Kansas, Minnesota, Missouri, Nebraska, North Dakota, Ohio, South Dakota, Utah, and Wyoming) primarily in the Midwest and Mountain West regions of the U.S. for years 2000 to 2019. The data were obtained from Departments of Transportation or Departments of Public Safety in each state. Data were not attainable for all states for all years, but the maximum number of years available for each state were included. The years of crash data included in this study for each state is listed in the Appendix, Table 5. State driver license renewal policies were manually extracted from state code.

Drivers aged 40 and older involved in fatal, injury, or property damage only crashes occurring between 2000 and 2019 were included. Vehicle occupants other than drivers and people not in the vehicle were not part of the study. Occupants of self-driving vehicles were also not included in this study.

### Variables

The main exposures examined in this study include the following driver license renewal policies: license renewal period (years), in-person renewal (every time, every other time, some other frequency), vision testing required at renewal (yes, no), knowledge tests required at renewal (yes, no), and mandatory physician reporting laws (yes, no). The other main exposure examined was driver age, which was grouped as: 40–54, 55–64, 65–74, and 75 and older. The primary outcomes examined included driver crash and driver injury rates per 100,000 population. Crashes with an injury were defined as crashes with the driver experiencing a minor, serious, or fatal injury as identified in the state crash report data. Possible injuries were not included in the definition of injury crash because of the subjective nature of possible injuries that could lead to greater misclassification of injury status. Population by age, sex, state, and year were determined with American Community Survey (ACS) Data accessed from the Integrated Public Use Microdata Series. Single year ACS datasets were used to enumerate resident population for each state for every year of crash data received from a state (Table 5) from 2000 to 2019 included [15].

A priori, crash rate models included covariates for drunk driving laws (law enforceable at blood alcohol content < = 0.08% and > 0.08%), unemployment rate (median cut point across study period, 4.4%), and year. Additional covariates assessed for potential confounding included: state traffic safety factors (maximum highway speed limits, seat belt laws, seat belt use rates) and environmental and community factors (per capita income, gasoline prices, and rurality). No additional covariates were identified as meaningfully confounding the relationship between state driving laws and crash rates (> 20% change in rate ratio).

State traffic safety factors were drawn from state-based traffic safety code and annual seat belt use surveillance reports from the National Highway Traffic Safety Administration [16–19]. Annual unemployment rates and per capita income data were drawn from the Bureau of Labor Statistics and the Bureau of Economic Analysis [20, 21] as a proxy for driving exposure. Gasoline price data were gathered from the US Energy Information Administration for each state [22]. Gasoline price, unemployment, seat belt use, and income data were all gathered by state and year. Four-level (urban, large rural city/town, small rural town, isolated small rural town) Rural Urban Commuting Area (RUCA) coding was used to categorize rurality of each crash location. The coordinates of crash locations were used to join crash data with maps containing the RUCA categories at a census tract level and then converted to county-level. For crashes missing coordinates, county was used. RUCA-level state population estimates for the denominator by age and year were adjusted by the proportion of population living in those county-level RUCA areas.

### Statistical analysis

Frequency distributions were examined for all license renewal policies. Negative binomial models were built using generalized estimating equations with an auto-regressive correlation structure and a log link function to allow for computation of rate ratios (rates per person) for in-person renewal and renewal length. Models were not built for vision testing, on-road driving test, knowledge test, or mandatory physician reporting policies due to lack of heterogeneity in those policies between states and across years. The models accounted for repeated measures from each state by using Generalized Estimating Equations clustered at the state level. Each law’s presence was assessed by quarter year periods and required to be present for the entire quarter before changing the exposure status. One main set of models included data from all 13 states. Two additional subsets were examined: 1) a subset of data from states that had a license renewal policy change (n = 7) and 2) the subset of states that had a license renewal policy change with the addition of a 5-year lag computed (n = 5). Crash data from 2005- to 2019 were matched to law data from 2000 to 2014 for the 5-year lag models. The 5-year lag considered a driver’s exposure to a law five years prior to account for the impact a law may have on a driver by removing or reducing their driving privileges at a younger age. For example, stricter in person renewal procedures may remove a driver in their 60 s and therefore result in a lower crash risk for that driver when they are 5 years older. This effect would be underestimated if the state had stricter requirements for drivers before 70 but licensing requirements like other states for drivers over 70. In other words, the 5-year lag also accounts for the lag in the time a law takes effect until a driver is impacted by the law change, which can sometimes take years.

The impact of the time to in-person renewal was assessed by constructing a variable that multiplied renewal length by in-person renewal requirement cadence (i.e., 1 for every renewal and 2 for every other renewal).

## Results

There were 19,010,179 drivers aged 40 and older involved in 12,538,424 unique crashes between 2000 and 2019 included in this analysis (Table 1). Among this population, slightly over half (54.3%) were aged 40 to 54 and about one-fifth (20.4%) were aged 65 and older, and 58.3% were male. For the large majority (85.1%), these drivers were not injured in the crashes studied. However, 1.6% were seriously injured and 0.2% had fatal injuries.

**Table 1:** Distribution of crash and driver characteristics, 13 states, 2000–2019

Variable	Level	N = 19,010,179	%
Driver age	40–54	10,325,239	54.3
	55–64	4,795,974	25.2
	65–74	2,402,936	12.6
	75 +	1,486,030	7.8
Crash year	2000–2005	3,149,762	16.6
	2006–2010	5,482,820	28.8
	2011–2015	5,526,773	29.1
	2016–2019	4,850,824	25.5
State blood alcohol limit	> 0.08	1,295,106	6.8
	< = 0.08	17,715,073	93.2
Unemployment rate	< = 4.4%	5,386,292	28.3
	> 4.4%	13,623,887	71.7
Driver sex	Male	10,427,948	58.3
	Female	7,472,236	41.7
	*Missing*	*1,109,995*	-
Driver injury severity	No injury	14,556,111	85.1
	Possible injury	1,309,568	7.7
	Minor injury	936,604	5.5
	Serious injury	267,605	1.6
	Fatal	40,367	0.2
	*Missing*	1,899,924*	-

*Injury severity was not present in the Wisconsin crash data provided (n = 1,441,507, 75.9% of missing data)

### Driver licensing laws

None of the 13 states had a mandatory physician reporting law during the study period (Table 2). Most states did not require knowledge tests (85%) at renewal for any age group. Driving tests were also rare with 92% of states not requiring a driving test at renewal for 40- to 74-year-olds and 85% not requiring a driving test for those aged 75 or older.

**Table 2:** Distribution of driver licensing laws by age group, 2010* vs. 2019

	2010								2019							
	Age Group								Age Group							
	40–54		55–64		65–74		75 +		40–54		55–64		65–74		75 +	
	N	%^‡^	N	%	N	%	N	%	N	%	N	%	N	%	N	%
**On-road driving test required at renewal**																
No	12	92	12	92	12	92	11	85	12	92	12	92	12	92	11	85
Yes	0	0	0	0	0	0	1	8	0	0	0	0	0	0	1	8
Other^1^	1	8	1	8	1	8	1	8	1	8	1	8	1	8	1	8
**Knowledge test required at renewal**																
No	11	85	11	85	11	85	11	85	11	85	11	85	11	85	11	85
Other^2^	2	15	2	15	2	15	2	15	2	15	2	15	2	15	2	15
**In-person renewal frequency**																
Every time	8	62	8	62	8	62	11	85	4	301	4	31	6	46	10	77
Every other time	3	23	3	23	3	23	2	15	6	46	6	46	4	31	3	23
Less than every time	2	15	2	15	2	15	0	0	3	23	3	23	3	23	0	0
**Vision test required at in-person renewal**																
No	2	15	2	15	2	15	1	8	2	15	2	15.4	2	15.4	1	7.7
Yes	10	77	10	77	11	85	12	92	6	46	7	53.8	9	69.2	12	92.3
Every other renewal	1	8	1	8	0	0	0	0	4	31	3	23.1	1	7.7	0	0.0
Other^3^	0	0	0	0	0	0	0	0	1	8	1	7.7	1	7.7	0	0.0
**Mandatory physician reporting**																
No	13	100	13	100	13	100	13	100	0	13	100.0	13	100.0	13	100.0	13
**Renewal period (Years)**																
2	0	0.0	0	0.0	0	0.0	1	7.7	13	0	0.0	0	0.0	0	0.0	1
3	0	0.0	0	0.0	0	0.0	1	7.7	0	0	0.0	0	0.0	0	0.0	1
4	5	38.5	5	38.5	6	46.2	6	46.2	0	5	38.5	5	38.5	6	46.2	6
5	5	38.5	5	38.5	5	38.5	4	30.8	3	5	38.5	5	38.5	5	38.5	4
6	2	15.4	2	15.4	1	7.7	0	0.0	5	2	15.4	2	15.4	1	7.7	0
8	1	7.7	1	7.7	1	7.7	1	7.7	3	1	7.7	1	7.7	1	7.7	1

*2010 was chosen as the comparison year in this table instead of 2000, given 2010 is the first year all 13 states were contributing data (see Appendix)

‡Some percentages may not add up to exactly 100 due to rounding

^1^NE: No test unless you fail vision test

^2^IL: Driver must take a written test every 8 years unless they have a clear driving record; UT: A 25 question open book written knowledge test may be required if you have had more than six citations in eight years

^3^Driver only has to undergo a vision test when renewing in person which is once every three years

Most states do require vision tests at every in-person renewal, but this decreased over time in the younger age groups. For example, 77% of the study states required 40–54-year-olds to take a vision test at every in-person renewal in 2010 compared to only 46% in 2019. Whereas, 92% of the study states required vision test at every in-person renewal for drivers age 75 and older in both 2010 and 2019.

**Fig. 1:**
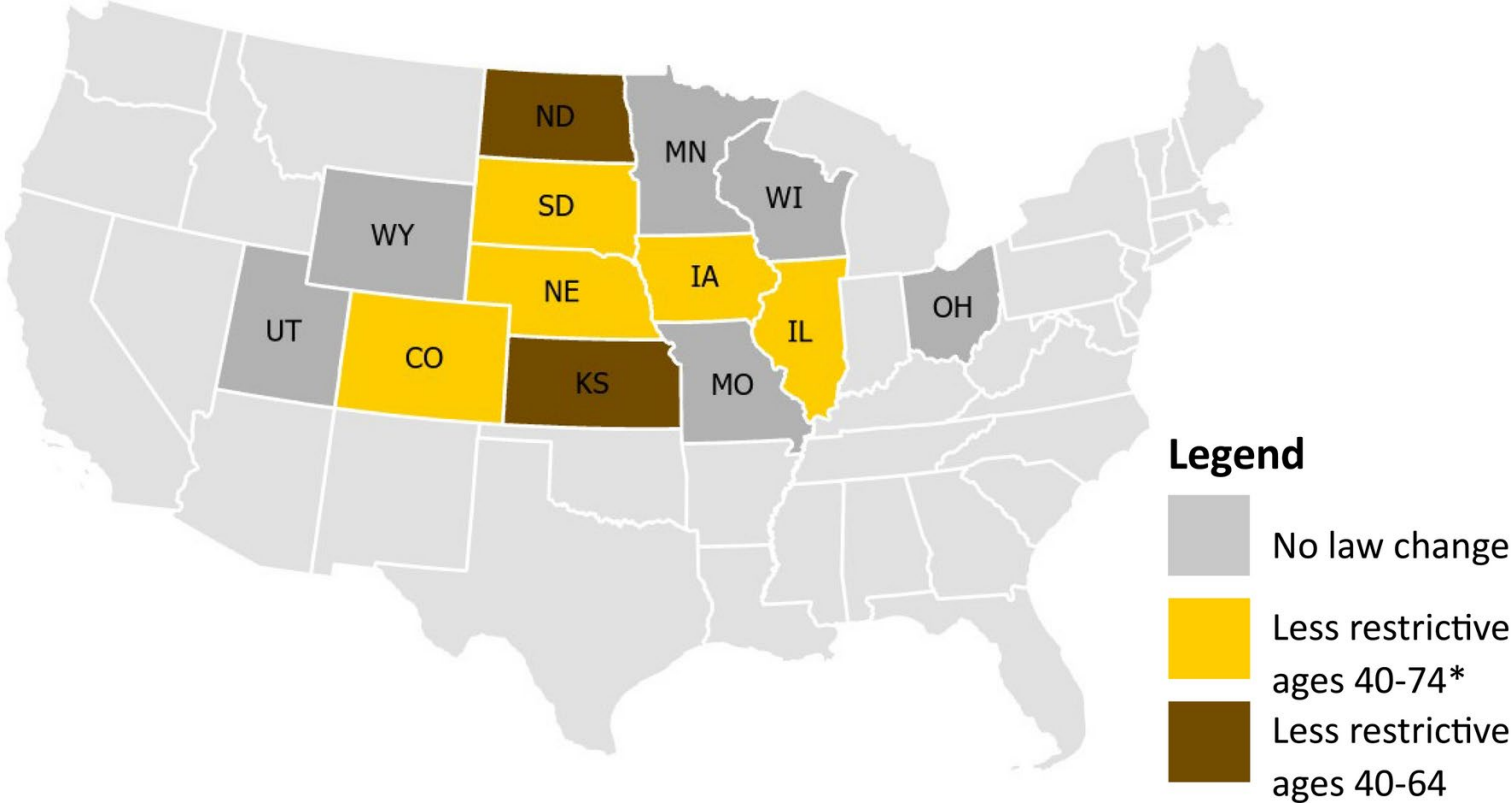
In-person renewal and/or renewal period law changes, 2000–2019. *40 + for South Dakota

The largest changes in licensing laws were among in-person renewal frequencies and renewal periods. During the study period, 7 of the 13 study states made changes to their in-person renewal and/or renewal periods, all of which were in the direction of loosening the requirements (Fig. 1; e.g., allowing more online renewals). Renewal periods increased across all age groups over the study period, even among the 75 and older age group. For example, 39% of the study states had renewal periods of 5 years or more for drivers aged 75 and older in 2010 versus 54% in 2019, meaning the times between having to renew a license increased even among the oldest drivers during the study period.

### Association between licensing laws and *crash* rates

License renewal laws that required drivers aged 65 to 74 to come in-person less frequently than every other time were associated with increased crash rates compared to in-person renewal being required every time (RR = 1.08, 95% CI: 1.01–1.16; Table 3). License renewals for the younger age groups (40–54 and 55–64) showed a similar association, though not statistically significant at the 95% confidence level. In-person renewals that were required every other time showed little difference from requirements of every time across all of the age groups. For drivers aged 75 and older, all states required in-person renewal either every time or every other time, so other less frequent in-person renewal requirements could not be examined.

**Table 3:** Associations between in-person renewal frequency and **crash** rates by driver age group, 2000–2019

Age Group	40–54	55–64	65–74	75 +
**All 13 states, comparing different levels of restrictiveness**				
**In-person renewal**	RR (95% CI)	RR (95% CI)	RR (95% CI)	RR (95% CI)
Every other time	0.99 (0.86–1.14)	1.01 (0.88–1.17)	1.03 (0.96–1.10)	1.03 (0.94–1.13)
Less frequent than every time	1.15 (0.97–1.37)	1.16 (0.99–1.36)	1.08 (1.01–1.16)	n/a
Every time	Ref	Ref	Ref	Ref
**Only states with a law change, model of less restrictive vs no change**				
	RR (95% CI)	RR (95% CI)	RR (95% CI)	RR (95% CI)
**Time to in-person renewal** Less restrictive^	0.92 (0.81–1.04)	0.92 (0.82–1.02)	1.02 (0.97–1.07)	1.11 (0.95–1.30)
**Only states with a law change, model of less restrictive vs no change, 5-year lag**				
	RR (95% CI)	RR (95% CI)	RR (95% CI)	RR (95% CI)
**Time to in-person renewal** Less restrictive^	0.98 (0.88–1.09)	0.97 (0.87–1.09)	0.96 (0.87–1.07)	1.07 (0.95–1.20)

*All models adjusted for: year, drunk driving laws, unemployment rate

^Less restrictive = change from every time to less than every time and/or longer renewal period

When narrowing the sample to only include states that had a law change during the study period, crash rates among drivers under less restrictive in-person renewal requirements did not differ greatly compared to those before the change (e.g., drivers aged 65–74 RR = 1.02, 95% CI: 0.97–1.07). However, among drivers aged 75 and older the crash rates were higher among drivers under less restrictive laws compared to drivers prior to the change, although not significant at the 95% confidence level (RR = 1.11, 95% CI: 0.95–1.30). Furthermore, when restricting the sample further to include only states that had a law change and accounting for a 5-year lag, results similarly showed little evidence of less restrictive in-person renewal requirements being associated with increased crash rates, but the rates were slightly elevated among the 75 and older age group (RR = 1.07, 95% CI: 0.95–1.20).

### Association between licensing laws and *injury* rates

License renewal laws that required drivers aged 75 and older to come in-person less frequently (time to in-person renewal less restrictive) were associated with increased injury rates compared to shorter renewal timing (RR = 1.10, 95% CI: 0.99–1.22; Table 4). Higher crash injury rates were observed with longer renewal periods for all other age groups, but the higher rates were not statistically significant. Furthermore, when narrowed to only states with a law change and factoring in a 5-year lag, the increased injury rate was not observed for any age group other than those over the age of 75 (RR = 1.18, 95% CI: 1.00–1.39).

**Table 4:** Associations between in-person renewal frequency and **injury** rates by driver age group, 2000–2019

Age Group	40–54	55–64	65–74	75 +
**All states, comparing if different levels of restrictiveness**				
**In-person renewal**	RR (95% CI)	RR (95% CI)	RR (95% CI)	RR (95% CI)
Every other time	1.01 (0.76–1.35)	1.05 (0.80–1.39)	1.09 (0.93–1.27)	1.03 (0.89–1.20)
Less frequent than every time	0.94 (0.63–1.41)	0.97 (0.68–1.38)	1.10 (0.93–1.30)	n/a
Every time	Ref	Ref	Ref	Ref
**Only states with a law change, model of less restrictive vs no change**				
	RR (95% CI)	RR (95% CI)	RR (95% CI)	RR (95% CI)
Time to in-person renewal Less restrictive^	1.10 (0.88–1.38)	1.08 (0.89–1.32)	1.13 (0.98–1.30)	1.10 (0.99–1.22)
**Only states with a law change, model of less restrictive vs no change, 5-year lag**				
	RR (95% CI)	RR (95% CI)	RR (95% CI)	RR (95% CI)
Time to in-person renewal Less restrictive^	1.02 (0.81–1.29)	1.01 (0.82–1.24)	0.93 (0.80–1.07)	1.18 (1.00–1.39)

^Less restrictive = change from every time to less than every time and/or longer renewal period

## Discussion

This study examined the effectiveness of driver license renewal policies on crash and injury rates over a 20-year period in 13 U.S. states. Generally, law changes observed in the study states resulted in longer times between license renewal, even for drivers aged 75 and older. Several states changed their policies around in-person renewal, allowing for more online renewal options. License renewal policy changes that were less restrictive (i.e., longer renewal period and/or less frequent in-person renewal) had some evidence of increased driver crash and injury rates. It is unclear why states tended toward loosening of restrictions, but the reasons are likely multifactorial, such as a combination of efforts to reduce administrative costs and improve efficiency and/or in response to demand from drivers.

Results from this study align with prior research showing more restrictive in-person renewal being associated with decreased fatality rates [13]. However, that prior evidence only showed such an effect in drivers aged 85 and older [13]. Conversely, prior research did not find an association between renewal period for drivers 55 or older, whereas in the current study a combined variable taking into account both renewal period and in-person renewal requirements found that less restrictive policies were associated with increased injury rates among drivers aged 75 and older.

Knowledge, vision, and driving test requirements varied little between states and none of the included states had mandatory physician reporting laws during the study period, so this study was not able to fully assess the impact of those license renewal policies on crash and injury rates. Prior research on mandatory road driving tests has been mixed, with one study showing decreased insurance claim rates and fewer drivers insured among drivers aged 75 and older [23], suggesting less driving, but other studies have not shown similar effects and an Australian study even found increased fatal and serious injury crash rates [24, 25].

Evidence from this and prior studies suggests that more restrictive in-person renewal requirements may contribute to reductions in injury and fatality rates among the oldest drivers. However, other license renewal policies (renewal period, knowledge tests, on-road driving tests, vision tests, and mandatory physician reporting) have limited and/or mixed evidence of effectiveness, suggesting that examination of alternative methods to assess fitness-to-drive and regulate driver licensure are likely warranted.

In addition to the common state driver licensure renewal policies examined in this study, states can impose licensure restrictions, and some use this method specifically to address older driver safety, such as placing restrictions to driving on local roads only, staying within a certain geographic area (e.g., within the town of residence), or not allowing driving after dark [4, 26]. Some states also have policies that subject drivers to additional testing following referral from a variety of sources such as referral from licensing personnel during a renewal following failure of a line exam, referral from police following a crash, or referral from a physician, family member, or even the driver themselves [4, 27, 28]. For example, in Iowa the most common referral sources are line exam and crashes, while physician, family member, and self-referral are rare [27]. However, regardless of referral source, once a driver enters the referral system they can be subjected to further testing to assess fitness-to-drive, such as cognitive tests including the Driver Orientation Screen for Cognitive Impairment (DOSCI) [29] and Safe Driving BASICS (Brief Auto-Screening Instrument for Cognitive Status) [27]. The DOSCI has been used to identify drivers with possible cognitive impairment, but not necessarily driving ability, while the tests included in Safe Driving BASICS (e.g., visual search, contrast sensitivity, hazard perception, information processing speed) have been developed to identify drivers with elevated crash risk [30–32].

In the U.S. driving is the preferred mode of transportation for the majority of older adults in all areas (urban, suburban, and rural) [7, 8, 33]. Driving is often the only viable transport option for those living in rural areas where transit and ride services are non-existent or highly limited [10, 11]. Given the dependence on driving, the ability to drive is highly correlated with older driver mobility and access to services which are often linked with connectedness and quality of life [9–11]. As such, driver licensing should ideally be aligned with fitness to drive, as opposed to seemingly arbitrary age cut-points or time periods that are common among state licensing renewal laws. Aligning licensure with fitness may also help to avoid unintended consequences license renewal laws such as premature driving cessation (i.e., removing a license too early) by older adults who are safe drivers because they are intimidated by or too nervous about the renewal process. Results from this study show some benefit of in-person renewal and frequency of renewal, but limited evidence of effectiveness of other renewal laws.

The lack of effect in many of the renewal laws suggests that finding alternative methods for conducting ongoing assessments of older drivers’ fitness to drive and driving performance may be more effective in removing unfit drivers from the road. Additionally, alternative methods such as cognitive screening can be administered based on individual driver referrals and reduce possible negative effects of premature driving cessation related to licensure renewal laws based on age cut-points. However, there is a lack of uniformity or widely accepted standard for assessing driving performance among older adults which needs to be addressed to inform road safety efforts [34]. Learning from and adapting performance-based approaches proposed for novice drivers may be one avenue to explore [35].

License renewal policies represent only one strategy for mitigating crashes and injuries among older drivers. While driver fitness is often scrutinized, it is crucial to recognize that not all crashes involving older drivers stem from driver fitness or ability limitations. There are many factors within the broader transportation system that contribute to insufficient road safety. Comprehensive approaches to improving road safety for older adults extend beyond licensing requirements and may include redesigning roadway infrastructure, transforming traffic safety culture, and increasing accessibility and adoption of alternative modes of travel such as public transit, bicycling, and walking.

### Limitations

This study has several limitations that should be noted. Not all of the 13 states contributed all years of data. However, our analytical design allowed for retaining all years of data, even if it did not cover the entire study period for each state. In addition to whole years of data missing in some states, there were also some missing data that were specific to certain states (e.g., Wisconsin was missing injury severity data and Colorado was missing driver sex data). As such, if a state was missing data for a certain sub-analysis the whole state had to be dropped.

Data used in this study was not able to account for driving exposure among the included drivers and instead relied on using population as the denominator (as opposed to miles traveled). This limitation was due to lack of data availability of miles traveled by age group and other possible denominators were similarly flawed or limited (e.g., number of licensed drivers) [36, 37]. As such, it was not possible to determine how much of the reduction in crash and injury rates observed in association with the in-person and renewal frequency were explained by things like drivers driving less or self-regulating (e.g., not driving after dark).

Additionally, the injury severity classifications relied on police-reported data which likely had between and even within states. Therefore, the ability to accurately measure injury outcomes likely varies. For example, some crashes reported as ‘possible injury’ may have resulted in an injury and should have been coded as ‘minor’ or ‘severe’ and would not have been included in our study. However, given the subjectivity that goes into injury severity of crash reports it is likely there is misclassification at nearly all levels. As such, we are unable to determine the directionality of possible bias stemming from these issues.

Despite the above limitations, this study provides useful information on impact of state driver licensing laws on crash and injury rates among older drivers that go beyond former work focused on fatality rates only and also provides updated data on these associations, given prior studies are primarily from a decade ago. Examining injury rates is particularly useful for understanding the potential burden of long-term effects of injuries that are often worse among older adults and can negatively influence their quality of life [38]. An additional strength of this study lies in the examination of multiple states, which enhances the generalizability of results.

## Conclusions

Loosening of driver license renewal laws showed increases in older driver crash and injury rates. Safety benefits of restrictive older driver licensing should be carefully weighted against costs to older driver well-being and quality of life resulting from licensure loss. Additional methods to assess fitness to drive could help to identify the mechanisms behind the increased crash and injury rates observed in this study related to less restrictive licensing laws and provide a more individualized approach to balance roadway safety with the prevention of premature driving cessation.

## Data Availability

The datasets used for the current study are available from the corresponding author upon reasonable request and within the constraints of the original data owners.

## Appendix

See Table 5.

**Table 5:** Years of crash data included in the study by state

CO	2007–2019
IA	2000–2019
IL	2007–2019
KS	2000–2019
MN	2004–2019
MO	2002–2019
ND	2005–2019
NE	2010–2019
OH	2000–2019
SD	2008–2019
UT	2010–2019
WI	2000–2019
WY	2000–2019
